# **Erratum for:** “Effect of gallium-arsenide laser, gallium-aluminum-arsenide laser and healing ointment on cutaneous wound healing in Wistar rats”

**DOI:** 10.1590/1414-431X2026e5949erratum

**Published:** 2026-08-31

R.V. Gonçalves, J.M.S. Mezêncio, G.P. Benevides, S.L.P. Matta, C.A. Neves, M.M. Sarandy, and E.F. Vilela

Correspondence: R.V. Gonçalves <reggysvilela@yahoo.com.br>

Erratum for Braz J Med Biol Res | ISSN 0100-879X (2010) 43(4): 350-355 | doi: 10.1590/S0100-879X2010007500022

The Authors notified the Editors of the Brazilian Journal of Medical and Biological Research that there was an error in Figure 1A, due to inappropriate processing with software calibration. The conclusion of the results remains unchanged. 

The correct Figure 1 is:

**Figure 1:**
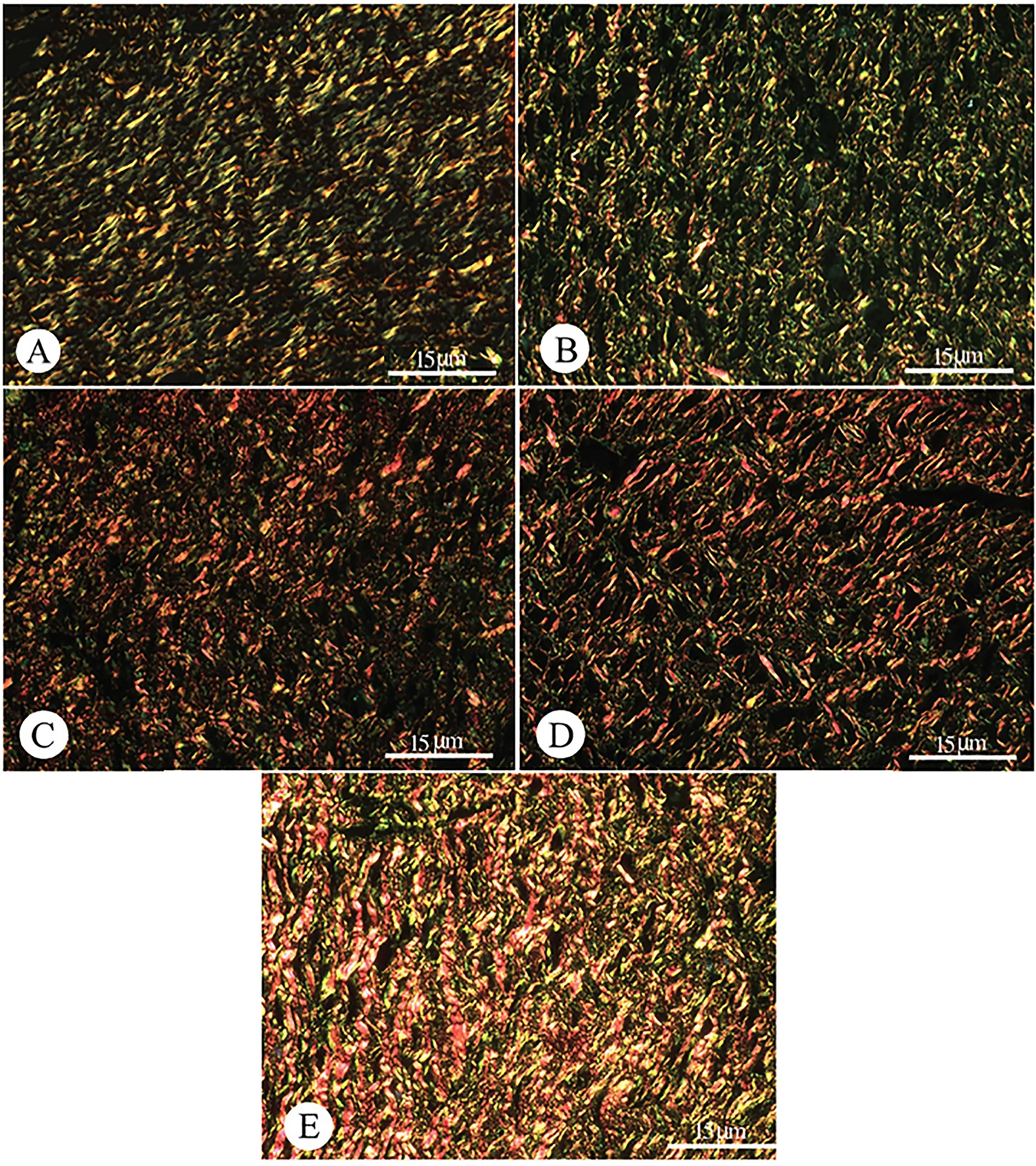
Histological sections of Wistar rat skin on the 20th day of the experiment stained with picrosirius and observed under a polarization microscope. *A*, Group 5 = control cleaned with 0.9% saline daily; *B*, group 4 treated with Dersani^®^ ointment; *C*, group 1 treated with gallium-arsenide laser (GaAs, 4 J/cm^2^); *D*, group 3 treated with gallium-aluminum-arsenide laser (GaAlAs, 60 J/cm^2^); *E*, group 2 treated with GaAlAs laser, 30 J/cm^2^. Groups 2 (L30) and 1 (L4) had a significant increase in collagen fibers compared with group 4 (D) and the control group (P < 0.05). Group 3 (L60) showed a significantly higher proportion of type I collagen fibers than group 4 (D; P < 0.05).

Editorial Team – Brazilian Journal of Medical and Biological Research

Received December 2, 2025; Accepted January 30, 2026; Corrected February 27, 2026

